# Prednisolone Versus Colchicine for Acute Gout in Primary Care (COPAGO): protocol for a two-arm multicentre, pragmatic, prospective, randomized, double-blind, controlled clinical trial of prednisolone and colchicine for non-inferiority with a parallel group design

**DOI:** 10.1186/s13063-023-07666-6

**Published:** 2023-10-05

**Authors:** Julia Truthmann, Julia Freyer Martins Pereira, Adrian Richter, Franziska Schuster, Amelie Witte, Susanne Böhm, Alexandra Greser, Petra Kamin, Sylvia Stracke, Marcus Dörr, Robin Bülow, Stefan Engeli, Ildikó Gágyor, Eva Hummers, Jean-François Chenot

**Affiliations:** 1 Department of General Practice, Institute for Community Medicine, University Medical Center Greifswald, Greifswald, Germany; 2 Department of Prevention Research and Social Medicine, Institute for Community Medicine, University Medical Center Greifswald, Greifswald, Germany; 3 Coordinating Center for Clinical Studies, University Medical Center Greifswald, Greifswald, Germany; 4 Department of General Practice, University Hospital Würzburg, Würzburg, Germany; 5 Department of General Practice, University Medical Center Göttingen, Göttingen, Germany; 6 Department of Internal Medicine, University Medical Center, Greifswald, Germany; 7 Institute for Radiology and Neuroradiology, University Medical Center Greifswald, Greifswald, Germany; 8 Institute of Pharmacology, University Medical Center Greifswald, Greifswald, Germany

**Keywords:** Primary care, Acute gout, Prednisolone, Colchicine, Pragmatic, Randomized controlled trial

## Abstract

**Background:**

Gout is the most common form of rheumatic disease in which monosodium urate crystals are deposited in the joints followed by acute inflammatory reactions. There are various approved drugs that can be prescribed for pain relief during an acute gout attack. However, to date, no direct comparison of efficacy of colchicine and prednisolone for the treatment of acute gout attacks has been investigated. Furthermore, the majority of previous research studies were not only conducted in tertiary centres but also excluded patients with common comorbidities due to contraindications to naproxen.

**Methods:**

This pragmatic, prospective, double-blind, double-dummy, parallel-group, randomized, non-inferiority trial investigates whether prednisolone (intervention) is non-inferior to treatment with colchicine (active control) in patients with acute gout. Adult patients presenting with acute gout to their general practitioners in 60 practices across 3 university sites (Greifswald, Göttingen, and Würzburg) are eligible to participate in the study. Participants in the intervention group receive 30 mg prednisolone for 5 days. Those in the control group receive low-dose colchicine (day 1: 1.5 mg; days 2–5: 1 mg). The primary outcome is the absolute level of the most severe pain on day 3 (in the last 24 h) measured with an 11-item numerical rating scale. Day 0 is the day patients take their study medication for the first time. They are then asked to fill out a study diary the same time each day for pain quantification. Pain scores are used for comparison between the two medications. Secondary outcomes are average response to treatment, swelling, tenderness and physical function of the joint, patients’ global assessment of treatment success, use of additional pain medication and non-pharmacological pain therapies. For safety reasons, potential side effects and course of systolic blood pressure are assessed.

**Discussion:**

This trial will provide evidence on the effectiveness of pain reduction and side effects of colchicine and prednisolone in acute gout in primary care.

**Trial registration:**

ClinicalTrials.gov Identifier: NCT05698680 first posted on January 26, 2023 (retrospectively registered). URL of trial registry record: https://clinicaltrials.gov/study/NCT05698680

**Supplementary Information:**

The online version contains supplementary material available at 10.1186/s13063-023-07666-6.

## Administrative information

Note: the numbers in curly brackets in this protocol refer to SPIRIT checklist item numbers. The order of the items has been modified to group similar items (see http://www.equator-network.org/reporting-guidelines/spirit-2013-statement-defining-standard-protocol-items-for-clinical-trials/).

**Table:** 

Title {1}	Prednisolone Versus Colchicine for Acute Gout in Primary Care (COPAGO): protocol for a two-arm multicentre, pragmatic, prospective, randomized, double-blind, controlled clinical trial of prednisolone and colchicine for non-inferiority with a parallel group design.
Trial registration {2a and 2b}.	ClinicalTrials.gov Identifier: NCT05698680
Protocol version {3}	Protocol version number: 2.0, 27.09.2022
Funding {4}	Funded by the German Federal Ministry of Education and Research.
Author details {5a}	Department of General Practice, Institute for Community Medicine, University Medical Center Greifswald, Greifswald, Germany Department of Prevention Research and Social Medicine, Institute for Community Medicine, University Medical Center Greifswald, Greifswald, Germany Coordinating Center for Clinical Studies, University Medical Center Greifswald, Greifswald, Germany Department of General Practice, University Hospital Würzburg, Würzburg, Germany Department of General Practice, University Medical Center Göttingen, Göttingen, Germany Department of Internal Medicine, University Medical Center, Greifswald, Germany Institute for Radiology and Neuroradiology, University Medical Center Greifswald, Greifswald, Germany Institute of Pharmacology, University Medical Center Greifswald, Greifswald, Germany
Name and contact information for the trial sponsor {5b}	University Medical Center Greifswald, Stefan Engeli: Stefan.Engeli@med.uni-greifswald.de
Role of sponsor {5c}	The funders were not be involved in the study design; collection, management, analysis, or interpretation of the data; writing of the report; or the decision to submit the report for publication. The sponsor (University Medical Center Greifswald) have ultimate authority over any of these activities.

## Introduction

### Background and rationale {6a}

Gout is one of the most common rheumatic diseases, affecting 3–6% of men and 1–2% of women in western countries [1]. In Germany, 1–2% of adults suffer from gout [2]. Due to the severe pain and impaired quality of life, the individual burden of disease during an acute gout attack is very high [3]. Currently, there are several approved medications available for the treatment of acute gout attacks. The European League Against Rheumatism (EULAR) guideline [4] recommends colchicine as the drug of first choice for acute gout attacks. According to the guideline, non-steroidal anti-inflammatory drugs (NSAIDs) and systemic corticosteroids can also be used. In contrast, the German Society for General Practice and Family Medicine (DEGAM) recommends prednisolone [5]. Prednisolone and low-dose colchicine were selected for this study because their effectiveness and safety profiles have not previously been directly compared.

Most commonly, acute gout attacks are treated in general practices. However, most previous studies have been conducted in specialized centres [4] and thus in a selective patient group. In addition, recent studies on the treatment of acute gout excluded approximately 20–30% of patients due to contraindications for NSAIDs [6, 7]. The absolute or relative contraindications include cardiovascular disease requiring antiplatelet treatment, oral anticoagulation, chronic kidney disease or a history of gastrointestinal disease.

The gold standard for the diagnosis of gout in rheumatology centres is the detection of monosodium urate crystals in aspirated joint fluid [8]. In primary care, the diagnosis of gout is made on the basis of clinical symptoms alone. Given the risk of injury and infection, joint puncture is usually not performed on patients in general practice. Additionally, microscopy with polarized light to identify urate crystals is not widely available in ambulatory care.

The dual-energy computed tomography (DECT) is offered to all participants as an optional examination to detect monosodium urate crystals in the affected joint. The amount of monosodium urate crystals (volume) is an indicator of disease burden.

### Objectives {7}

This non-inferiority trial aims to investigate whether prednisolone (intervention) is noninferior or inferior within acceptable limits to treatment with colchicine (active control) in acute gout in primary care. Both treatments are compared based on the improvement in absolute pain scores achieved on day 3 after initiation of drug therapy. Secondary outcomes are average response to treatment, swelling, tenderness and physical function of the joint, patients’ global assessment of treatment success, use of additional pain medication and non-pharmacological pain therapies. For safety reasons, potential side effects and course of systolic blood pressure are assessed.

### Trial design {8}

The study “Prednisolone Versus Colchicine for Acute Gout in Primary Care” (COPAGO study) is a multi-centre, pragmatic, prospective, double-blind, double-dummy, parallel-group randomized non-inferiority trial comparing two approved treatments for acute gout—prednisolone and colchicine (phase IV). The study has two arms of active and effective treatments. Allocation to the treatment arms is 1:1, and study participants are randomized to either the prednisolone or colchicine group. To ensure blinding, both treatment arms receive a placebo in addition to the effective drug. All investigators and the study statistician are blinded to the randomization list.

## Methods: participants, interventions and outcomes

### Study setting {9}

Patients presenting with an acute gout attack are being recruited across 60 general practices and 3 university study sites in the Greifswald, Göttingen and Würzburg area in Germany. The central project management is the Department of General Practice of the University Medical Center Greifswald. The Institute of General Practice of the University Medical Center Göttingen and the Department of General Practice of the University Hospital Würzburg are study sites and act as local study coordination centres for the respective regions of Göttingen and Würzburg. The complete list of study sites can be obtained on ClinialTrials.gov.

### Eligibility criteria {10}

Inclusion criteria:

- Adult patients ≥ 18 years of age
- Acute onset (existing since the previous day at the most) of pain in hand or foot (podagra, chiragra)
- Clinical diagnosis of acute attack of gout (symptoms: pain, swelling, tenderness, redness or local hyperthermia)
- Willingness to participate in the study and ability to give written informed consent

Exclusion criteria:

- Known intolerance or contraindication to either medication
- Known intolerance to the placebo (e.g. lactose intolerance)
- Existing or less than 2 weeks ago oral treatment with corticosteroids or colchicine
- Known chronic kidney disease (CKD stage 4 or greater) or an available value of estimated glomerular filtration rate (eGFR) < 30 ml/min/1.73 m.^2^
- Known haematopoietic disorder or available values of platelets < 30,000 μl or leucocytes < 4000 μl, or Hb < 5 mmol/l/ or 8 g/dl
- Uncontrolled high blood pressure (systolic blood pressure permanently above 160 mmHg)
- Known liver cirrhosis or severe liver disease or available liver enzymes results (i.e. serum glutamate oxalate transaminase (SGOT) and serum glutamic pyruvic transaminase (SGPT)) being elevated by more than twice the respective reference range
- Known current gastric or duodenal ulcer (diagnosed in the last 4 weeks)
- Current chemotherapy or chemotherapy completed less than 3 months ago
- Known human immunodeficiency virus (HIV) infection
- Solid organ transplant with immune suppression
- Desire to have children within the next 6 months in both men and women
- Existing pregnancy or breastfeeding
- Participation in other studies according to the German Medicines Act in the last 3 months
- Participation in the COPAGO study with past gout attack

### Who will take informed consent? {26a}

The patient is informed about the clinical study by their general practitioner (GP). Patients receive the patient information and sufficient time for reflection regarding study participation. If desired, patients may return to the waiting room to read the patient information. Patients are encouraged by their GP to ask any questions they have about study participation during the consultation. Before the patient can be included in the study, written consent for participation and data processing must be provided via the informed consent form. This consent can be withdrawn at any time.

### Additional consent provisions for collection and use of participant data and biological specimens {26b}

Study participants have the option to undergo a voluntary DECT examination at the radiology department of the University Medical Center Greifswald, Göttingen or Würzburg within 14 days after study inclusion. During their initial visit at their GP’s practice, the patients can indicate the wish to participate and consent can be withdrawn at any time. Imaging of both feet is performed using a Siemens Dual Source SOMATOM Definition Flash or SOMATOM Force. Prior to the DECT examination, the participants are once again informed about potential risks.

## Interventions

### Explanation for the choice of comparators {6b}

The EULAR guideline recommends colchicine as the drug of first choice for acute gout attacks. According to this guideline, NSAIDs and systemic corticosteroids can also be used. In contrast, DEGAM recommends using prednisolone. The need for a randomized controlled clinical trial comparing colchicine and corticosteroids has been highlighted by various studies [4, 6, 9]. Unlike most studies conducted in tertiary care centres, this study is based in primary care and meets the recommendations of the EULAR guideline [4] for future research. In previous studies in primary care, patients with common comorbidities, such as cardiovascular disease, oral anticoagulation, chronic kidney disease or a history of gastrointestinal disease, were excluded due to contraindications to naproxen [6, 7] or the studies were unblinded [6]. The present study includes patients from primary care with comorbidities and is therefore more representative than previous trials [4].

The dosing of the study drugs is based on the recommendations of the EULAR [4] and the DEGAM guideline [5]. Unlike in the USA where colchicine is available in 0.6 mg tablets, in Germany, only 0.5 mg are available. Both drugs are administered in tablet form. The study design is double-blinded and randomized to prevent the influence of physician treatment preferences regarding the use of prednisolone or colchicine in acute gout on study data. Due to the different intake regimen, placebos are used in addition to the effective drug (double-dummy method).

### Intervention description {11a}

The intervention arm receives prednisolone 30 mg and a placebo and the active control arm is treated with colchicine 0.5 mg and a placebo. See Table 1 for an overview of the interventions.

**Table 1:** Study medication plan

	**Medication**	**Timing**	**Day 0**	**Day 1**	**Day 2**	**Day 3**	**Day 4**
**Intervention arm**	Prednisolone (30 mg)	am	1 tablet	1 tablet	1 tablet	1 tablet	1 tablet
	+						
	*Placebo*	*am*	*2 tablets*	*1 tablet*	*1 tablet*	*1 tablet*	*1 tablet*
		*Evening*	*1 tablet*	*1 tablet*	*1 tablet*	*1 tablet*	*1 tablet*
**Active control arm**	Colchicine (0.5 mg)	am	2 tablets	1 tablet	1 tablet	1 tablet	1 tablet
		Evening	1 tablet	1 tablet	1 tablet	1 tablet	1 tablet
	+						
	*Placebo*	*am*	*1 tablet*	*1 tablet*	*1 tablet*	*1 tablet*	*1 tablet*

The study medication is manufactured by Tiofarma B.V. (Oud-Beijerland, Netherlands). Both active drugs are licenced products. In addition, placebo tablets of similar appearance and composition but without an active ingredient are manufactured by this company. The study participants receive a treatment consisting of two tablets: one with an active substance and one that is a placebo that is identical in taste and appearance to the corresponding drug (the prednisolone placebos contain a bittering agent to ensure a taste similar to the active drug). In this double-dummy design, participants in both study arms receive the same number of tablets and drug prediction is more difficult. Participants randomized to the active control arm receive colchicine plus prednisolone placebo and participants randomized to the intervention arm receive prednisolone plus a colchicine placebo.

The optional DECT examination is offered to participants in both trial arms to detect the presence and amount of monosodium urate crystals in both feet. Since the joints of the feet are the main site of manifestation of acute gout attacks, crystal deposits in the feet are also expected in participants with a gout attack in other joints. In order to ensure comparability of the volume measurement, the DECT examination is therefore limited to the feet. The feet were scanned using commercial CT scanners (Siemens Somatom Force or Definition Flash).

### Criteria for discontinuing or modifying allocated interventions {11b}

The criteria for the discontinuation or modification of allocated interventions are: the prescription of drugs which are contraindicated in combination with study medication in the context of a medical emergency as determined by the treating physician, a serious adverse event between day 0 and 4 (see the “Adverse event reporting and harms {22}” section) or an unscheduled visit of the participant due to persistent severe pain. Study participants receive a study ID card that identifies them as participants in the COPAGO clinical trial and are asked to carry it with them for the duration of the study. Among other information, their study ID number and an emergency number for the study sponsor is provided on the card.

If drugs that are contraindicated in combination with colchicine or prednisolone have to be prescribed in an emergency, the treating physician can ask for unblinding using the aforementioned emergency number and the participant can stop taking the study medication. If rescue medication due to persistent pain is required, the study protocol recommends prescribing prednisolone as additional pain medication (according to the DEGAM guideline [5]), regardless of allocated intervention and blinding is maintained. All participants who discontinue a trial intervention will continue to be followed up, unless they withdraw consent.

### Strategies to improve adherence to interventions {11c}

The trial participants document intake of the trial medication in the study diary. The patients are required to attend their GP’s practice for a second visit between days 6 and 8 of the study. Here, the study participants are required to return the packaging and blisters of the study medications and the study diary. The return of the investigational medicinal products and the pill count is documented in the Drug Accountability Log.

### Relevant concomitant care permitted or prohibited during the trial {11d}

During the trial, GPs are encouraged to only prescribe the study medication and no other pain medication. Patients are informed that the study medication they receive is a licensed medication to treat an acute gout attack and are encouraged to only take the study medication. If participants take additional pain medication for any reason or are prescribed prednisolone on a rescue basis during study participation, this must be documented in the study diary.

### Provisions for post-trial care {30}

If necessary, individual post-trial care is offered by the treating GP. The expected rate of serious adverse events caused by the trial drugs is very low as they are approved medications, which are used on-label and their administration is based on guideline recommendations.

### Outcomes {12}

The primary and secondary end points were defined according to the recommendation of the Outcome Measures in Rheumatoid Arthritis Clinical Trials Group (OMERACT) [10–12]. However, little evidence is currently available on the reliability and validity of these commonly used patient reported outcomes in gout research. Evidence on the Pain Numeric Rating Scale (NRS, 0–11) is based on a single, unpublished study. The NRS seems to have face, content and construct validity and is sensitive to changes within and between groups in gout patients [13]. No studies on the validity of the outcomes of joint swelling, joint tenderness, patient global assessment and activity limitation are available.

#### Primary outcome

The primary outcome is the efficacy of prednisolone compared to low-dose colchicine measured as most severe pain in the last 24 h on day 3 on an 11-point  NRS (0 stands for “no pain” and 10 for “the strongest pain imaginable”) and compared across treatment groups.

Previous studies indicate a significant reduction in pain between days 2 and 5 of an acute gout attack, even without treatment [11, 14]. In a study by Roddy et al. [6], mean pain decreased by approximately 50% from day 0 to day 3 with treatment of naproxen or colchicine. Therefore, the most severe pain in the last 24 h measured at day 3 of treatment was chosen as the primary endpoint. To ensure comparability, the average response to treatment from days 1 to 6 of follow-up will be analysed as a secondary outcome.

#### Secondary outcomes

- 1. Average response to treatment: most severe pain in the last 24 h on an 11-point numerical rating scale across treatment days (from day 1 to 6 of follow-up) compared across treatment groups.
- 2. Reduction in joint swelling and tenderness: measured using 4-point Likert scales. Swelling is quantified as “no joint swelling”, “palpable”, “visible”, and “bulging beyond the joint margins”. Tenderness (sensitivity to touch) of the joint is quantified as “no pain”, “pain”, “pain and winces”, and “pain, winces and withdraws”. Treatment groups are compared on day 3 of treatment.
- 3. Physical function of the joint: measured on an 11-point numerical rating scale (0 indicating “not at all/no problem” and 10 indicating “worst pain ever”) using the following questions:
  - How much are you now restricted in your normal daily activities by the gout attack?
  - How much trouble do you have putting on a shoe today? (for participants with podagra only)
  - How much pain do you have when you are walking today? (for participants with podagra only)
  - How much trouble do you have grasping and holding something with your affected hand? For example, when unscrewing a bottle. (for participants with chiragra only)
    Values on day 6 compared to baseline are considered across treatment groups.
- 4. Patients’ global assessment of treatment success: measured with a 5-point Likert scale as: “excellent”, “very good”, “good”, “fair”, and “poor” on day 6 after baseline and compared across treatment groups.
- 5. Most severe pain in the last 24 h depending on disease duration: measured on an 11-point NRS (as described for the primary outcome). The model will be adjusted for disease duration.
- 6. Frequency of use of additional pain medication by treatment group: assessed as type of pain medication, dose and reason for intake. The frequency of use of additional pain medication between baseline and day 6 per treatment group will be compared.
- 7. Frequency of use of non-pharmacological pain therapies: assessed as the application of cooling or immobilization/elevation of the affected joint. The frequency of use of non-pharmacological pain therapies per treatment group will be compared.

### Other outcome measures (DECT examination)

- 1. Presence and volume of monosodium urate crystals in both feet: presence and volume of crystals (ml) will be determined by trained personnel based on imaging data.
- 2. Associations between crystal volume and patient characteristics (e.g. age, sex, previous gout attacks).
- 3. Associations between crystal volume and use of uricostats/uricosurics (yes/no).
- 4. Associations between crystal volume and most severe pain at baseline.

#### Safety

- 1. Type and frequency of side effects: assessed as dizziness, nausea, vomiting, dyspepsia, diarrhoea, constipation, abdominal pain, headache, skin rash or other self-reported symptoms.
- 2. Frequency and severity of serious adverse events: assessed as any event which results in death, is life-threatening or requires inpatient hospitalization (as defined in current legal bases).
- 3. Course of systolic blood pressure (baseline to day 6): a baseline measurement takes place in the GP`s practice. If the patient has his or her own blood pressure device, a daily independent measurement is performed at home from day 1 to day 6.

### Participant timeline {13}

**Fig. 1:**
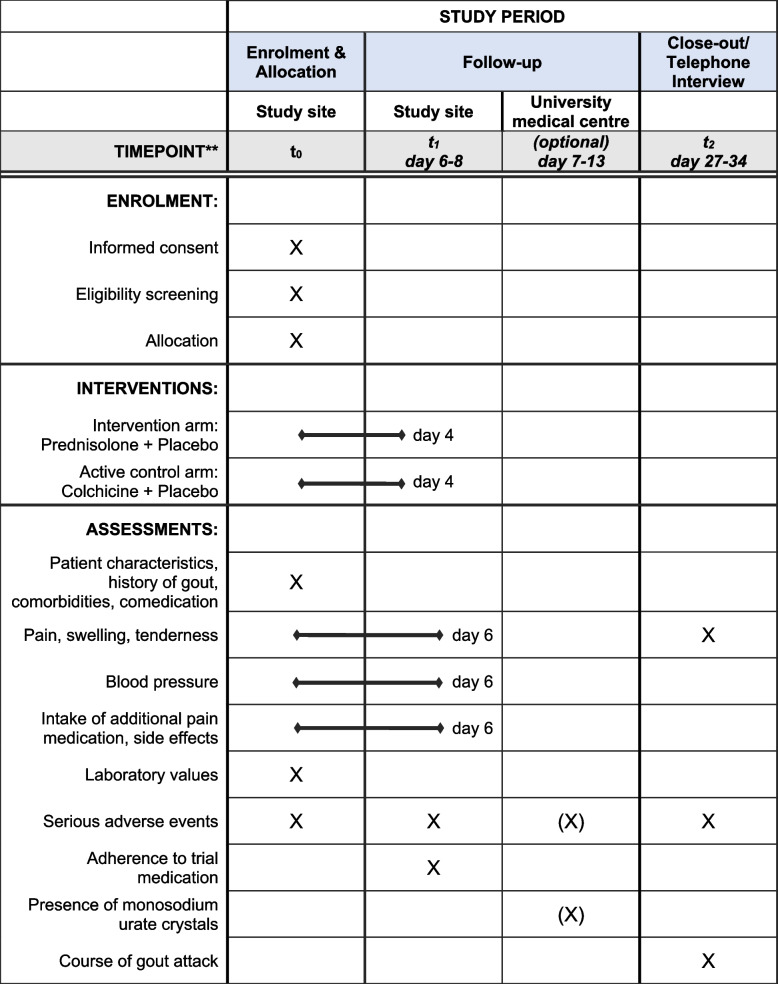
Study schedule of enrolment, interventions, and assessments. X: assessment at specific timepoint. (X): optional assessment at specific timepoint. Interventions (line): medications are taken from day 0 to day 4. Assessments (line): outcomes assessed from day 0 to day 6

**Fig. 2:**
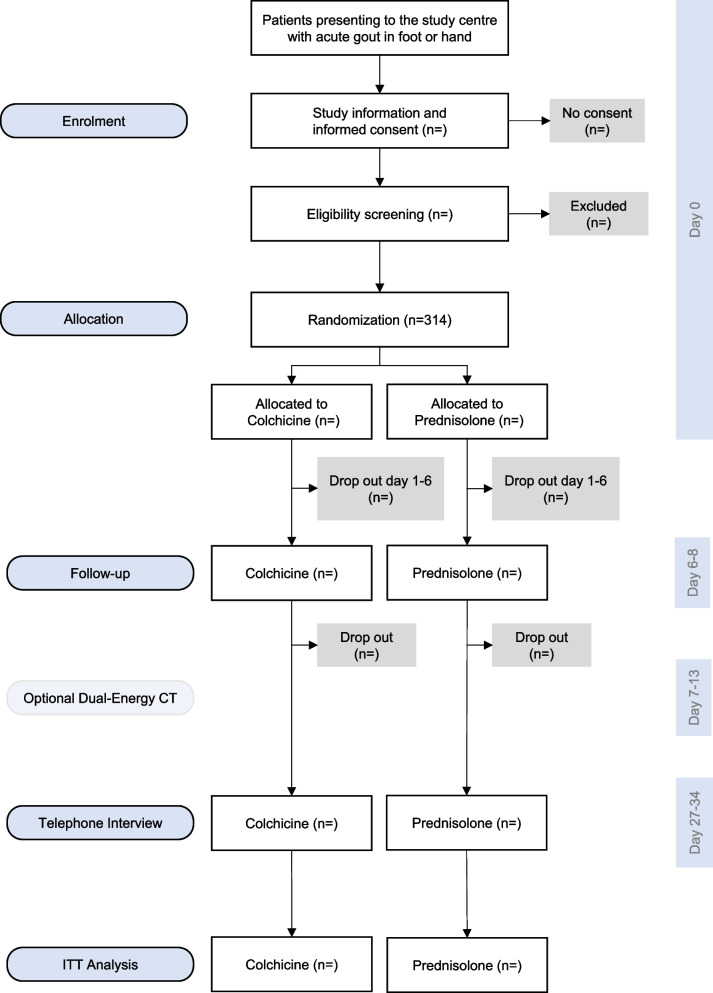
Participant flow diagram according to Consolidated Standards of Reporting Trials (CONSORT)

The participant timeline is illustrated in Figs. 1 and 2 (patient`s version provided as Additional file 1). This clinical trial includes 2 visits to the patient’s GP’s practice (at baseline and on days 6–8), an optional visit for a DECT examination at the university medical centre in their respective study region (days 7–13) and a telephone interview on days 27–34. The study period for the individual participant is 4 weeks.

On day 0, patients with an acute gout attack in the hand or foot present to their GP. If gout is confirmed, patients provide consent and are eligible for participation in the study; they are randomly assigned to one of the two treatment groups; both drugs are administered for 5 days and both treatment groups receive a placebo (double-dummy method), so that neither the patient nor the GP know the allocation arm. Blood samples are taken and a laboratory test is performed to determine serum uric acid levels, as well as inflammatory markers and renal function. The patients are asked to complete a questionnaire and, among other things, are requested to quantify the most severe pain they have experienced in the last 24 h on a numerical rating scale of 0–10.

During days 1 to 6, patients are asked to fill in a study diary. The primary and secondary outcomes (pain, joint swelling, joint tenderness) and, if further analgesia is needed, the use of additional pain medication are recorded in the diary. Participants, who have a blood pressure monitor, are asked to measure and record their blood pressure daily. On day 6, the patients are also asked to assess potential functional limitations caused by the gout attack and to give a global assessment of the treatment success.

After 1 week, patients attend their GP’s practice for a follow-up visit (visit 2). They are asked to return their study diary and (empty) medication packets.

After 4 weeks, the patients are contacted via telephone by our study nurses and asked about the clinical course of their gout attack (recurrence of an acute gout attack, further treatment, duration of incapacity to work, adverse events). The telephone call lasts about 15 mi.

In addition, study participants receive the offer to have a one-time dual-energy CT (DECT) examination of their feet on days 7–13 to check for the presence of uric acid crystals. Images are taken of both feet.

### Sample size {14}

A power analysis was conducted using PROC POWER of SAS version 9.4 (SAS Institute Inc., Cary, NC, USA). Based on the minimal clinically important difference in pain (primary outcome measured on a 11-point NRS), the non-inferiority margin was set to ${\delta }_{NI}=1$ [15]. Previous studies of prednisolone vs. naproxen and naproxen vs. colchicine suggest that the difference of mean pain levels between prednisolone and colchicine will most likely not exceed 0.22 units [6, 7]. Based on these assumptions with a 90% power and a significance level of $\alpha =5$, the sample size required would be 142 participants per arm. To account for 10% expected dropout, a total sample size of 314 (157 per trial arm) is needed.

### Recruitment {15}

The aim is to recruit 314 patients across 60 general practices and 3 coordinating university clinics over 2.5 years. Patients who have been diagnosed with gout in the previous 2 years were identified by the study GPs from their electronic medical records. These patients receive an information letter notifying that their general practice is taking part in the COPAGO study and offer their patients to participate in the study should they have another acute gout attack. This approach is chosen so that information about the study can be provided in advance and patients have longer to think about potential study participation. This procedure is analogous to the study by Roddy et al. [6].

Furthermore, posters and leaflets providing information regarding the study are displayed in each participating general practice and more information can also be found online (including an information video).

## Assignment of interventions: allocation

### Sequence generation {16a}

Participants will be randomized in a 1:1 ratio to the trial arms. A restricted randomization in random blocks using the R package blockrand is applied. We expect a heterogeneous recruitment rate between centres and dropout of single study sites.

### Concealment mechanism {16b}

The trial medication for the study sites is sequentially numbered with a patient identification number. Participants are assigned to the patient identification numbers in ascending order. Allocation concealment is ensured, as the allocation is not visible for the teams of the study sites.

### Implementation {16c}

The study statistician created the R script for the generation of the randomization list, but the setting of the seed is conducted by a statistician who is part of the sponsor`s team. The principal investigators enrol participants and assign them to interventions.

## Assignment of interventions: blinding

### Who will be blinded {17a}

Participants and recruiting GPs as well as study staff at the university study sites are blinded to treatment allocation.

### Procedure for unblinding if needed {17b}

If unblinding is required, the treating physician can contact the sponsor team of the trial via the 24/7 emergency number provided. Unblinding is then accomplished via an online tool which is accessible only to the sponsor team. The following information is assessed: reason for unblinding, patient identification number, name and function of the person requesting unblinding.

## Data collection and management

### Plans for assessment and collection of outcomes {18a}

All data are recorded in paper form. Data collection forms comprise a patient questionnaire, a GP case report form, a patient diary, an optional dual-energy CT case report form and a telephone interview questionnaire. All data collection forms are available from the corresponding author on request.

As this is a pragmatic study conducted in general practices, blood samples are analysed in the affiliated laboratories of the practices.

The dual energy examination takes place in the three university hospitals by trained staff. The procedure of the examination and the measurement parameters are defined in a standard operating procedure. To assess the quality of the reading, two trained radiologists will review a subset of the images independently. Inter- and intra-observer reliability will be determined.

### Plans to promote participant retention and complete follow-up {18b}

Patients are informed that both drugs are licensed to treat acute gout attacks and both medications are used for relieving pain, thus reassuring them that no matter which treatment group they are allocated to, they receive active-ingredient medication. Furthermore, patients receive self-care guidelines on what to additionally do to help treating their pain and be reassured that, if required, they can return to the general practice any time for an additional visitation. If required, rescue medication can be prescribed.

To promote participants retention, patients can only receive reimbursement for their participation when they have returned their study diary and completed the second assessment at the study site (t_1_).

### Data management {19}

**Fig. 3:**
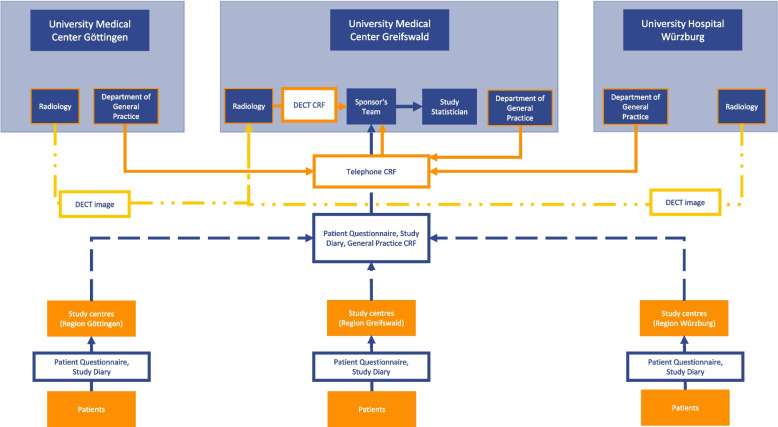
Data flow

Data is collected on a handwritten basis. The investigators and all study team members are trained to comply with clinical trial documentation requirements. The flow of the study data is presented in Fig. 3. Patient questionnaires and study diaries are completed by the participants. During the second assessment at the study site, the investigator checks completeness of the data. The GP case report form is completed by the investigator (GP) and trained practice staff. The optional DECT examination is conducted at the three university study sites, but reading of the images and completion of the DECT case report form is performed by radiologists at the University Medical Center Greifswald. The telephone case report form is completed by study staff at the university study sites. A member of the sponsor team (the monitor) verifies the accuracy of the data and the consistency with the source data during on-site monitoring visits. Data entry is completed by trained staff and verified via double entry. Inconsistent data entries are resolved through a consensus process that is supervised by the sponsor team. Electronically captured raw data will represent a 1:1 correspondence to paper-based data. To derive pre-processed analysis data, data curation will be done using SAS scripts. The database will be locked after the last patient has completed all visits according to the study schedule. The data will be stored on servers of the University Medical Center Greifswald.

### Confidentiality {27}

Personal data of enrolled participants (name and phone number) is collected only for contacting purposes throughout the trial (e.g. telephone interview and optional DECT examination). The principal investigator forwards the data to the university study sites via fax. After the telephone interview is completed, the telephone number is deleted. For all further purposes, participants receive a pseudonym. Prior to giving informed consent, participants are informed in detail about data management and data protection in this trial. No patient sensitive data are sent to the sponsor`s team.

### Plans for collection, laboratory evaluation and storage of biological specimens for genetic or molecular analysis in this trial/future use {33}

Blood samples are collected in the study sites and analysed in the affiliated laboratories. After the analysis, blood samples are discarded.

## Statistical methods

### Statistical methods for primary and secondary outcomes {20a}

#### Primary analysis

A multiple linear regression model will be computed to analyse whether prednisolone is non-inferior to colchicine regarding pain at day 3. In addition to the group allocation, the model will be adjusted for pain at baseline, age and sex.

#### Secondary analyses

Linear mixed effects models will be fitted for the average level of pain (secondary outcome 1) and physical function at day 6 (secondary outcome 3). A two-sample Wilcoxon rank sum test will be applied for the outcomes joint swelling, joint tenderness and patients’ global assessment of the treatment effect (secondary outcomes 2 and 4). Most severe pain depending on disease duration (secondary outcome 5) will be analysed using the model of the primary analysis but with adjustment for disease duration instead of age. Frequencies and confidence intervals of addition pain medication use and non-pharmaceutical pain therapies will be presented. If these therapies are used in both treatment arms, multiple logistic regression models adjusted for treatment arm and baseline pain will be used.

### Interim analyses {21b}

No interim analysis will be conducted.

A premature termination or suspension of this clinical trial can be initiated by the sponsor/principal investigator, the Federal Institute for Drugs and Medical Devices, the higher federal authority and the ethic committees. In the event of premature termination or interruption by the sponsor/principal investigator, the sponsor/principal investigator will report and justify the decision to the Federal Institute for Drugs and Medical Devices and the ethic committees stating the reasons for the termination or interruption. In the case of an interruption, the sponsor will make any effort to continue the clinical trial to the regular termination.

Early termination of the clinical trial will be considered if:

- The risk–benefit ratio of intervention and control drug for the trial participants changes significantly,
- New scientific findings show that a continuation is no longer reasonable,
- Prescription of one of the study drugs is no longer justifiable,
- The sponsor deems it necessary to discontinue the trial for safety reasons (e.g. on the advice of the data safety monitoring board, DSMB),
- The clinical study is no longer feasible,
- Early evidence of superiority or inferiority of a treatment group is provided by an interim evaluation or other research results.

### Methods for additional analyses (e.g. subgroup analyses) {20b}

The primary analysis will be stratified according to the following categories:

- Elevated uric acid vs. normal or low uric acid level
- Elevated CRP vs. normal CRP level
- Use of pain medication prior to inclusion (yes vs. now)
- Presence of crystal deposits measured by DECT (yes vs. no)

### Methods in analysis to handle protocol non-adherence and any statistical methods to handle missing data {20c}

The primary outcome will be analysed according to the intention-to-treat principle. Multiple imputation will be used to handle missing data and ensure analysis of all patients.

### Plans to give access to the full protocol, participant level-data and statistical code {31c}

Anonymized versions of the datasets analysed during the current study will be made available upon reasonable request.

## Oversight and monitoring

### Composition of the coordinating centre and trial steering committee {5d}

The coordinating centre of the trial is composed of the Department of General Practice and sponsor team at the University Medical Center Greifswald and the local university study sites in Göttingen and Würzburg. The clinical trial coordination team oversees the execution and management of the trial and ensures exchange between all stakeholders. This team conducts weekly meetings and provides daily support for the study. There is no steering committee for the study.

### Composition of the data monitoring committee, its role and reporting structure {21a}

An independent committee (data safety monitoring board) ensures safety and data quality by:

- Considering recruitment rates and problems,
- Ensuring protocol adherence,
- Monitoring reports including amount and seriousness of findings,
- Advising whether to continue, modify (protocol amendments) or stop the trial,
- Investigating necessities of protocol amendments, and
- Reviewing adverse events, SAEs and SUSARs.

The committee is composed of independent board members including: a statistician, a GP, a rheumatologist and a clinical pharmacologist. The trial committee members conduct telephone conferences in a regular manner. The committee reports to the sponsor team and the coordinating investigator as appropriate.

### Adverse event reporting and harms {22}

All adverse events that occur during the study are recorded. Patients document new symptoms or complications in their study diaries. GPs review these entries during the second assessment at the study site and assess severity, intensity and causality. Adverse events are additionally assessed documented at the optional DECT examination (days 7–13) and during the telephone interview (days 27–34).

In case of any serious adverse event (SAE), study investigators are to inform the sponsor team within 24 h. We assume a high level of patient safety because both drugs are licensed for the treatment of acute gout and the duration of treatment is short.

### Frequency and plans for auditing trial conduct {23}

Oversight and supervision of the clinical trial is documented by the monitor, who acts on behalf of the sponsor. The monitor is authorised to inspect the source data (e.g. data from laboratory diagnostics, medical records) for comparison with the case report forms. He or she verifies that all collected data are accurate and that the safety and the rights of all subjects are guaranteed. Data are monitored centrally.

The monitoring process is based on a plan provided by the sponsor. To ensure a high quality of data collection, the sponsor team provides SOPs to harmonize all major processes. In addition, templates are provided to ensure a complete documentation of the clinical trial according to GCP. After the inclusion of the first patients, the first monitoring visit serves to identify any difficulties in completing the case report forms or other deviations from the study protocol. All following monitoring visits are to be conducted in a risk-adjusted manner. The monitoring visits are conducted by the monitor with assistance of the corresponding study nurse (Greifswald, Göttingen or Würzburg). Moreover, study nurses conduct additional site visits on demand to assure quality. After the termination of the trial, a close-out visit will be conducted.

### Plans for communicating important protocol amendments to relevant parties (e.g. trial participants, ethical committees) {25}

When and where amendments are required, they will be reported to the authorities and ethic committees without delay, and after approval, the investigators will be informed.

### Dissemination plans {31a}

The results of this study will be made available to the scientific community by publishing the findings in scientific journals and on ClinicalTrials.gov as well as to the broader public by communicating the results on websites, social media and in the press.

## Discussion

There is a lack of evidence for first-line treatment of acute gout attacks. The results of this pragmatic randomized controlled trial will help to make more specific recommendations for treatment of acute gout with prednisolone compared to colchicine.

Most gout patients are managed in primary care. Previous studies were mainly conducted in specialized centres and thus comprised a selective patient group with a higher disease burden. As we conduct a pragmatic trial in general practices, we expect this study to have a high external validity and the results can be easily generalized to primary care. The pragmatic approach affects the following design aspects of the study:

### Inclusion criteria

In contrast to previous trials, patients with frequent comorbidities, e.g. cardiovascular diseases, may participate in the study. In addition, the diagnosis of acute gout is based on clinical presentation alone as recommended in the DEGAM guideline [5]. Due to associated risk of injury, bleeding and infection, aspiration of synovial fluid is not performed in primary care practices and will not be used in the present study to confirm the diagnosis of gout.

### Interventions

Prednisolone and colchicine are both licensed drugs for treatment of acute gout and will be used on-label. Because prednisolone and colchicine have different intake regimens, a double-dummy technique is needed to maintain blinding.

### Primary and secondary outcomes

Efficacy is defined by self-reported pain at day 3 and also the secondary outcomes are patient-reported. Two trained patient research partners of the German Rheumatism League (Deutsche Rheuma-Liga Bundesverband e.V.) were actively involved in the planning phase of the trial (e.g. patient reported outcomes, trial flow), discussed study materials (e.g. informed consent, information material) and will be engaged in the interpretation and dissemination of the results. Thus, we expect trial results, which will be highly relevant to participants.

Based on evidence from previous randomized controlled trials allowing an indirect comparison of prednisolone and colchicine [6, 7], we do not expect major differences in treatment effects. The results of this trial will contribute to guidelines to make more specific recommendations for first and second choice drugs for treatment of acute gout with regard to comorbidities (contraindications) and side-effects.

The aim of the optional DECT examination is of an explanatory nature. A subgroup analysis will examine the primary outcome in persons with and without crystal deposits in the feet. In addition, the amount of monosodium urate crystals in the joint, which is an indicator of disease burden, will be described in patients recruited in primary care. Previous DECT studies are small and primarily involve people with long-standing, established disease [16]. This descriptive analysis of crystal volume provides the basis for designing future studies to analyse the efficacy of uric acid-lowering therapy in primary care.

## Trial status

Protocol version number: 2.0, 27.09.2022; Recruitment started on January 18, 2023. The end of recruitment will be approximately February 28, 2025.

### Supplementary Information

**Additional file 1.** Trial flow for patients.

## Data Availability

Anonymized versions of the datasets analysed during the current study will be made available upon reasonable request.

## Abbreviations

CKD: Chronic kidney disease
COPAGO: Prednisolone Versus Colchicine for Acute Gout in Primary Care
CRF: Case report form
DECT: Dual-energy computed tomography
DEGAM: German Society for General Practice and Family Medicine
DSMB: Data safety monitoring board
eGFR: Estimated glomerular filtration rate
EULAR: European League Against Rheumatism
GCP: Good clinical practice
HIV: Human immunodeficiency virus
NRS: Numerical rating scale
NSAIDs: Non-steroidal anti-inflammatory drugs
SGOT: Serum glutamate oxalate transaminase
SGPT: Serum glutamic pyruvic transaminase
SOP: Standardized operating procedure
