# Supplementary material for: Prednisolone Versus Colchicine for Acute Gout in Primary Care (COPAGO): protocol for a two-arm multicentre, pragmatic, prospective, randomized, double-blind, controlled clinical trial of prednisolone and colchicine for non-inferiority with a parallel group design
Source: Trials. 2023 Oct 5;24:643. doi: 10.1186/s13063-023-07666-6 (PMC10557330; doi:10.1186/s13063-023-07666-6)
Supplement: Supplementary file 1 — Additional file 1. Trial flow for patients. [file 13063_2023_7666_MOESM1_ESM.pdf]

Trial Flow

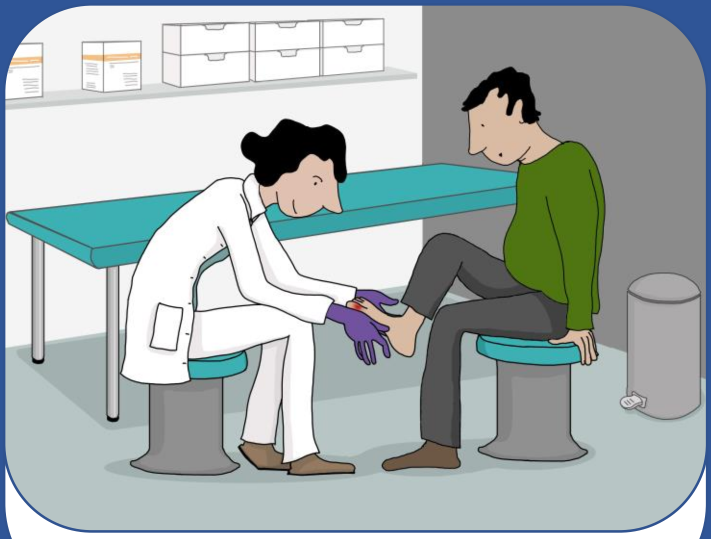

Examination

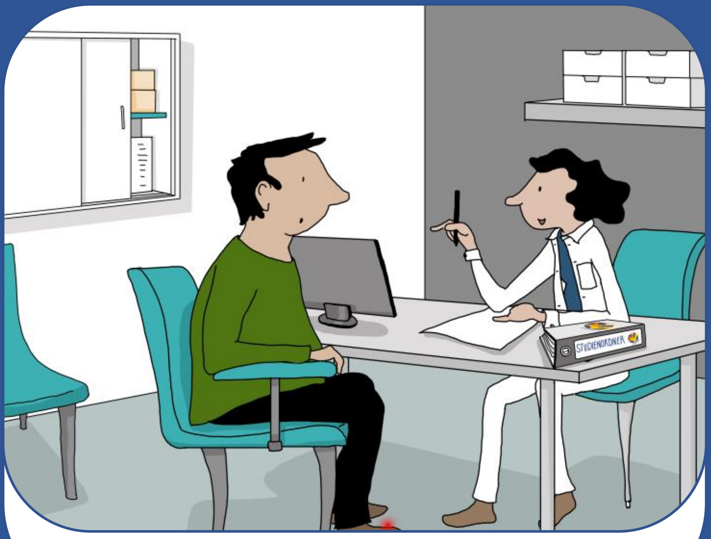

Study Consultation

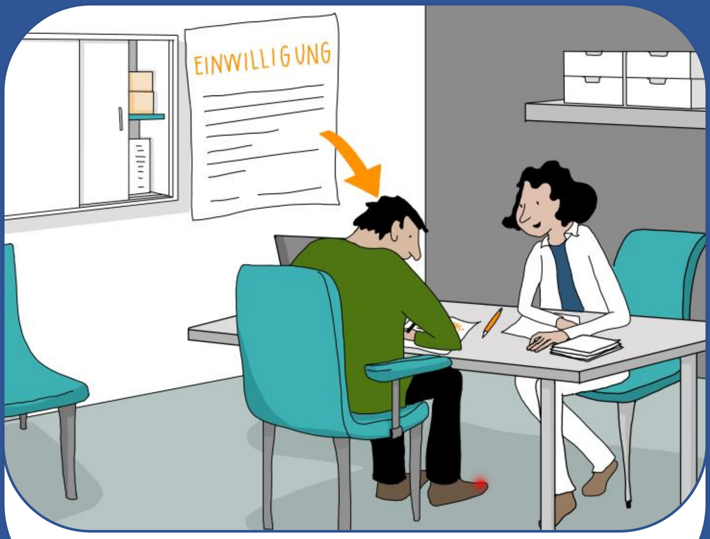

Informed Consent

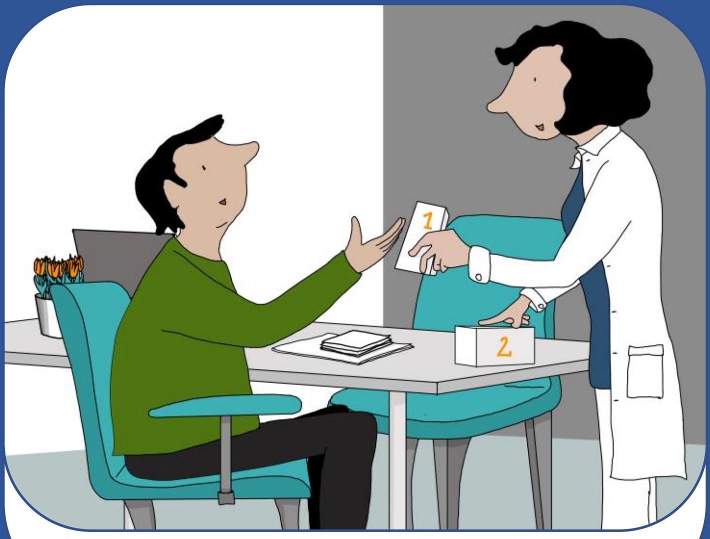

Handover of Medication

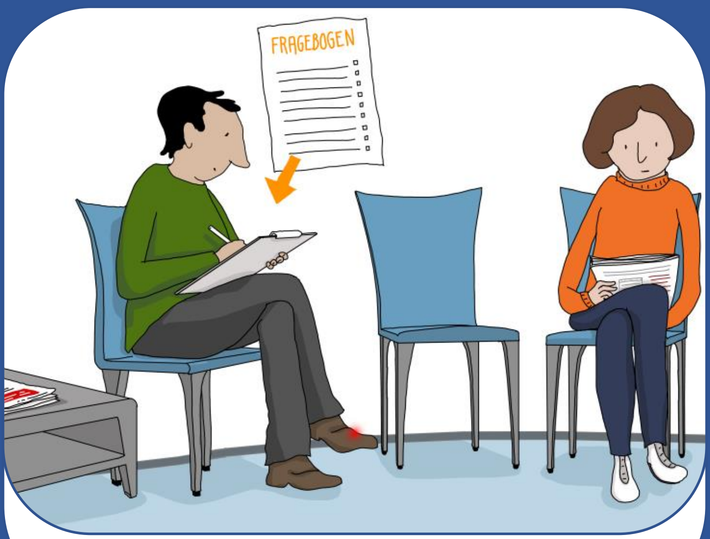

Completion of Questionnaire

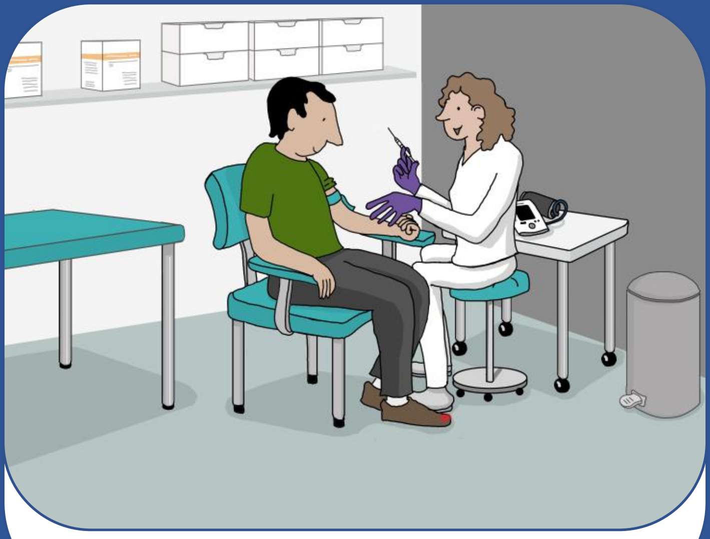

Collection of Blood Samples

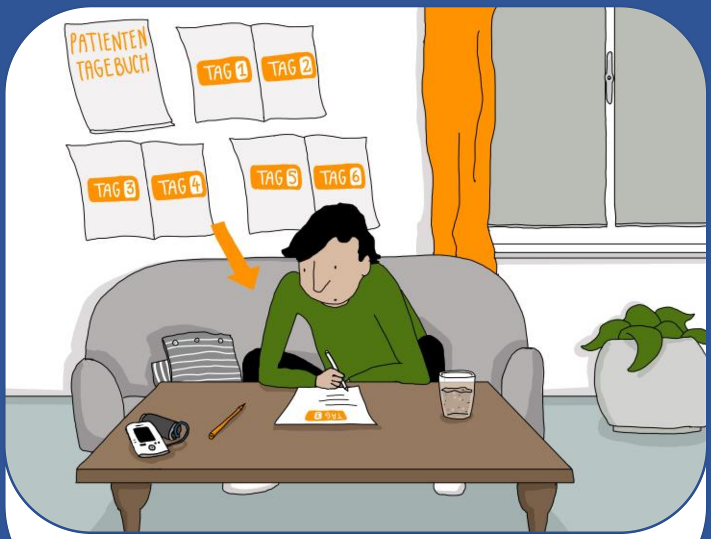

Daily Completion of Study Diary

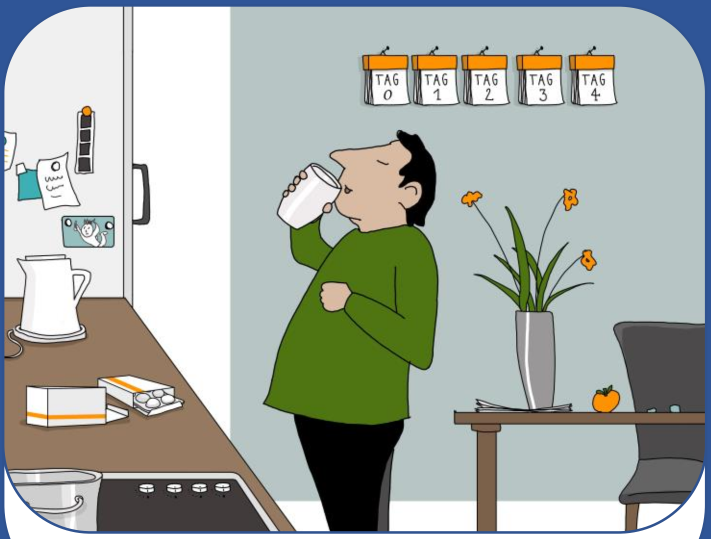

Daily Intake of Medication

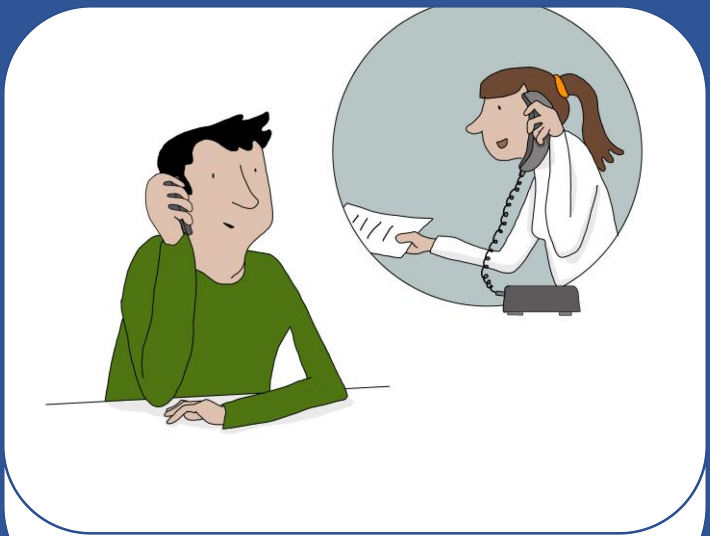

Optional: Schedule DECT Examination

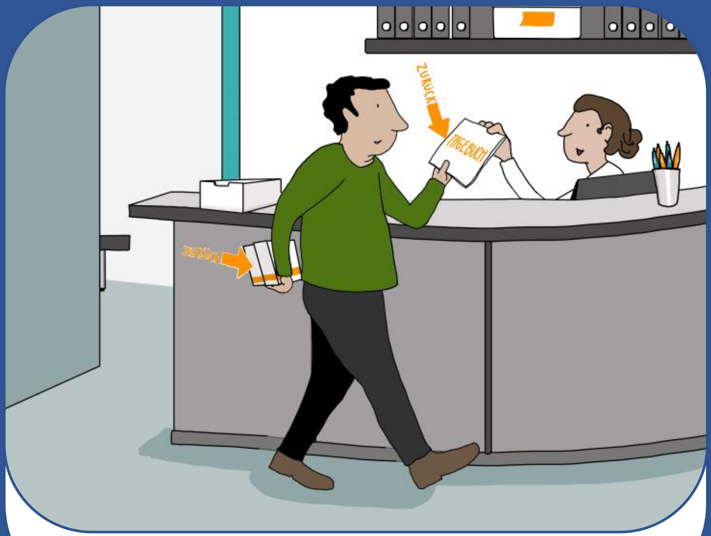

Return of Study Material

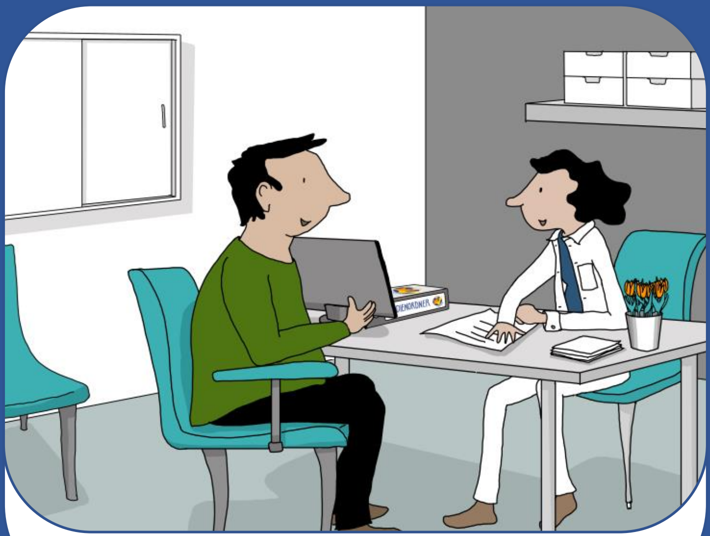

Final GP Appointment

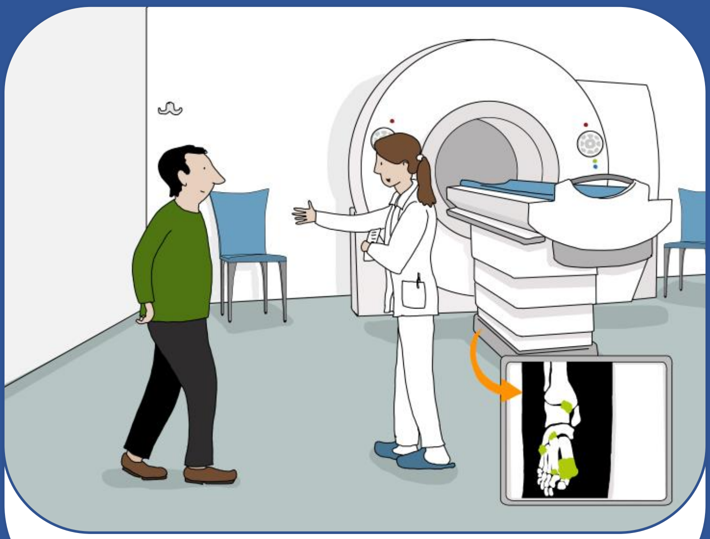

Optional: DECT Examination

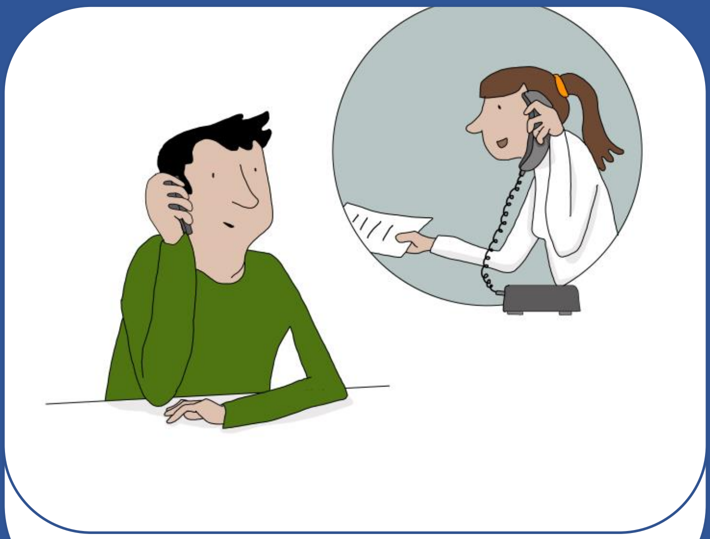

Telephone Interview

Dual Energy-CT:

The DECT examination is optional. This means you can participate in the COPAGO study without undergoing the DECT examination. If you opt for the DECT examination, the results will be sent directly to your GP. Please arrange a separate appointment with your healthcare provider for the evaluation.

Images: Marie Hübner (graphic telling), https://www.graphictelling.org
